# Revisiting the Modeling of the Conversion Gain of CMOS Image Sensors with a New Stochastic Approach

**DOI:** 10.3390/s22197620

**Published:** 2022-10-08

**Authors:** Gil Cherniak, Amikam Nemirovsky, Yael Nemirovsky

**Affiliations:** 1 Electrical Engineering Department, Technion—Israel Institute of Technology, Haifa 3200003, Israel; 2 Department of Electrical Engineering, Kinneret College on the Sea of Galilee, Tzemah 1513200, Israel

**Keywords:** CMOS Image Sensor, photon counting, low read noise, conversion gain, shot noise, pinned photodiode, global shutter

## Abstract

A stochastic model for characterizing the conversion gain of Active Pixel Complementary metal–oxide–semiconductor (CMOS) image sensors (APS) with at least four transistors is presented. This model, based on the fundamental principles of electronic noise, may provide a reliable calibration of the gain conversion, which is one of the most important parameters of CMOS Image Sensor pixels. The new model revisits the “gold standard” ratio method of the measured variance of the shot noise to the mean value. The model assumes that shot noise is the dominant noise source of the pixel. The microscopic random time-dependent voltage of any shot noise electron charging the junction capacitance C of the sensing node may have either an exponential form or a step form. In the former case, a factor of 1/2 appears in the variance to the mean value, namely, q/2C is obtained. In the latter case, the well-established ratio q/C remains, where q is the electron charge. This correction factor affects the parameters that are based on the conversion gain, such as quantum efficiency and noise. The model has been successfully tested for advanced image sensors with six transistors fabricated in a commercial FAB, applying a CMOS 180 nm technology node with four metals. The stochastic modeling is corroborated by measurements of the quantum efficiency and simulations with advanced software (Lumerical).

## 1. Introduction

Complimentary metal–oxide–semiconductor (CMOS) active pixel sensors (APS) with at least four transistors per pixel are currently the technology of choice for consumer electronics as well as scientific applications and emerging 3D imaging for specific use cases [1,2,3,4,5,6,7,8,9,10,11,12,13,14,15,16]. CMOS APS architecture may vary between different designs tailored to specific applications. The most common method of evaluation for image sensors is using optical input. However, the pixel and ADC characteristics cannot be evaluated separately in digital imagers. If separate evaluation is not possible, it is impossible to directly confirm whether any noise contribution is from the pixel or the ADC side. As a result, the characterization of Advanced CMOS imagers is quite challenging nowadays.

Raw data generated by modern digital camera systems are measured in the form of relative digital numbers, or DNs. The digital number DN is physically meaningless. Therefore, it is essential that a constant be found that converts DN units to absolute electron units.

The Conversion Gain (CG) for camera characterization has traditionally been determined with “gold standard” mean variance and/or photon-transfer techniques [17,18,19]. These methods break down when the sensor response is nonlinear, as is known to be the case with APSs. Since the pixel and ADC characteristics cannot be evaluated separately, the measured noise results cannot be attributed to the pixel or the ADC side. As a result, performance parameters may be estimated with high inaccuracies.

Moreover, the pixel should not be treated as a black box, as assumed in the past. A recent paper has demonstrated the need to relate the measured Conversion Gain with a specific Pixel Layout [20]. This paper exhibits several APS pixel designs, fabricated on the same die and implemented in the 180 nm CMOS Image Sensor (CIS) technology node with four metal layers, at a commercial FAB. The Conversion Gain is increased by reducing the Sensing Node Capacitance with local process variants, which reduce the parasitic capacitance (see Section 2) as well by redesigning the source follower within the design rules of the process.

The importance of digital CMOS Imagers keeps growing for many new use cases. The challenges of how to characterize the performance parameters of digital imagers are also growing, since the ADC and the pixels cannot be measured separately. The conventional approach of treating the system as a black box with well-defined conversion gains needs to be revisited.

The stochastic model presented in this work can be compared with two previous modeling approaches: physics-based modeling and statistical Monte-Carlo simulations.

(1) Electrostatic, physics-based models of the pinned photodiode (PPD) combined with the transfer gate are reported in [21,22]. A set of analytical expressions is derived for the 2D electrostatic profile, the PPD capacitance and the charge transfer current. The models are validated with technology computer-aided design simulations and stationary and opto-electrical simulations.
(2) A complementary statistical modeling approach is based on Monte-Carlo simulations [23,24].

The new stochastic model presented in this study has emerged from the need to characterize a state of-the-art CMOS imager with six transistors [25]. The quantum efficiency of the pixels has been modeled with the advanced software LUMERICAL [26], while the measurements were performed by a dedicated setup. The measured results, based on the conventional Conversion Gain, yielded high quantum efficiencies above the simulated ones, whereas it was expected that the measured results would be lower due to the photo-carrier’s losses. This is how the authors realized that the Conversion Gain needs to be revisited.

Accordingly, this paper revisits the definition of Conversion Gain and its measurement based on a new stochastic model. Section 2 briefly reviews the terms defining the conversion gain. These terms are applied to a state-of-the-art CMOS imager with 6T APS. Section 3 presents the stochastic model, while Section 4 presents measurements based on the modeling of Section 3. Section 5 presents the simulations, while Section 6 summarizes this study. The results are in line with previous papers [23,24]: “Whereas the literature and conventional wisdom have focused on either $\sqrt{kTC}$. or $\sqrt{kTC/2}$. like behavior, the noise is found to behave like shot noise for both small and large signals”. While in the past, this conclusion was based on Monte Carlo simulations, our stochastic model is based on fundamental physical considerations.

## 2. The Conversion Gain: A Key Parameter in Advanced CMOS Image Sensors (CIS)

For the sake of clarity for a wider community, we briefly review the basic schematic layout of a 4T CIS as well as the more advanced imager with 6T transistors that is under study [25].

**Figure 1:**
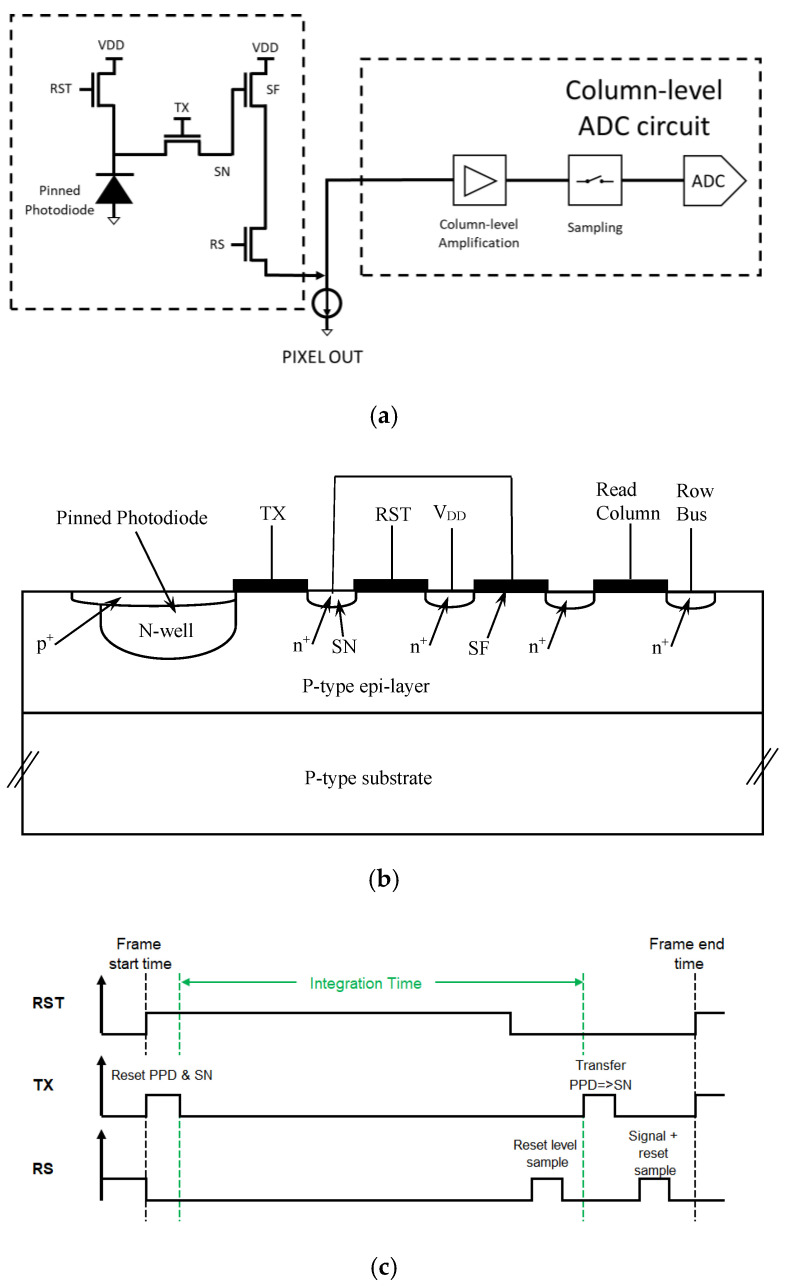
(**a**) A schematic overview of the pixel, marked by the dashed line, with its readout chain, marked by a separate dashed line. The column-level ADC circuit is presented schematically. (**b**) Schematic cross-section of the pixel. The Floating Diffusion (FD) of the Transfer Gate is the Sensing Node (SN), which is connected to the gate of the Source Follower. (**c**) The timing diagram showing how the thermal noise of the reset operation is reduced. The first sample measures the noise, while the second sample measures the same noise and the signal. By subtracting the two readings, this noise is canceled.

Figure 1 shows the schematic of a conventional 4T CIS readout chain based on a Pinned Photodiode (PPD), an in-pixel source follower stage and column-level amplification that are analyzed with correlated double sampling.

The four transistors are the Reset Transistor (RST), the Transfer Gate (TX), the Source Follower (SF) and the Row select (RS). Figure 1b is the cross-section of the 4T pixel. The different parasitic elements contributing to the sensing node (SN) capacitance include the overlap capacitances of the transfer and reset gates, respectively, the sensing node junction capacitance C and the parasitic capacitance related to the metal wires. These capacitances are independent of the in-pixel SF and they are added. By optimizing the SN doping (with implantation), the parasitic capacitance of the SN may be reduced [20].

Figure 1c is the timing diagram showing how true correlated double sampling is achieved.

In the above design, at least two conversions are required in the readout process: charge must be transferred from the PPD to the floating sense node (in-pixel) and then by the analog to the digital circuit (out-of-pixel).

The Sensor Conversion Gain is obtained by multiplying two fundamental conversion stages in an APS: the charge-to-voltage conversion *V*/*e* and the analog-to-digital conversion *DN/V* [17]. Accordingly, the Conversion Gain is defined as the number of electrons represented by each digital number, and it is denoted by *DN*/*e*.

(1) $$CG: [\frac{V}{e}]\cdot [\frac{DN}{V}]= [\frac{DN}{e}]$$

The analog conversion gain, usually defined in terms of microvolt per electron, is, in practice, determined by the sensing node capacitance, since V = Q/C. Conversion gains of the order of 160 [µV/e] or higher are considered high. The 160 µV per electron is obtained for a capacitance of ~1 [fF]. Physically, the conversion gain is related to the value of the capacitance C of the sensing node.

The specification for the conversion gain (high or low) is dictated by the application. Low-noise CMOS Image Sensors for low-light applications are designed with a high Conversion Gain. A high CG is the key for deep sub-electron noise pixels based on PPD and in-pixel SF. With a high CG, the contribution of noise from the readout, referred to the input, is less significant. However, the penalty is a smaller dynamic range. Pixels designed with a dual high- and low-gain feature have been demonstrated to enable low noise without a dynamic range penalty [27].

**Figure 2:**
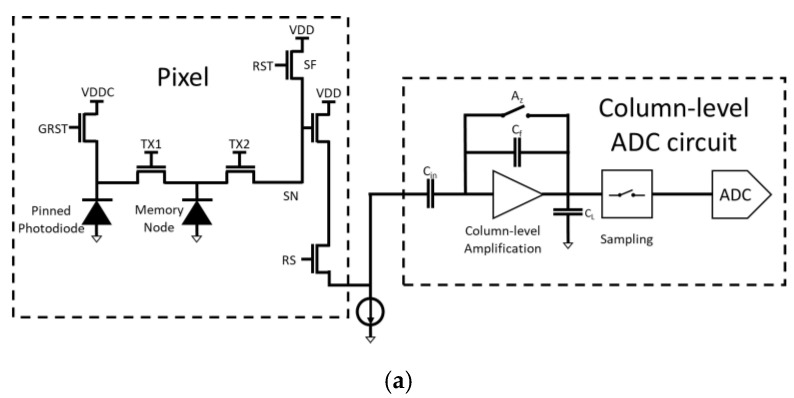

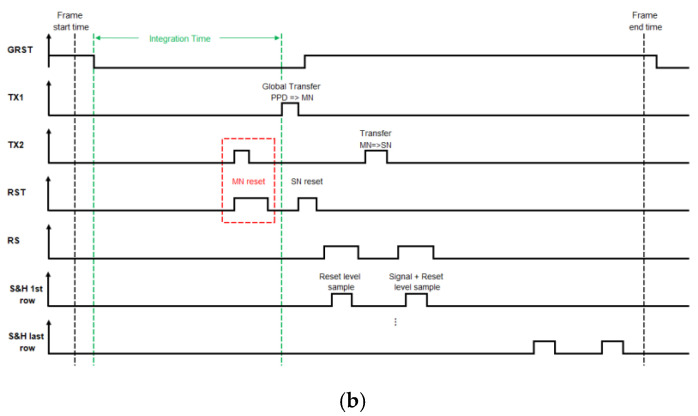
(**a**) Schematic presentation of a 6T APS, including the Global Reset Shutter (GRST), the transfer gate TX1 to the memory node (MN) and the additional transfer gate TX2 to the sensing node (SN). The reading of the signals with the source follower then follows exactly as in the four-transistor-APS (of Figure 1). The column-level ADC circuit is presented schematically. (**b**) Timing diagram of 6T APS, including the Global Shutter (GRST) and true Correlated Double Sampling (CDS).

The advanced CIS include six transistors [28], as shown in Figure 2. One more transistor is required for Global Shuttering. These pixels also need a memory node, and, hence, there is one more transfer gate. The memory node, a Floating Diffusion which provides a small (non-linear) capacitance, is implemented below the first transfer gate. Global Shuttering enables the induction of the same and simultaneous integration time for all the pixels. The signal photons are transferred from the Pinned Photodiode (PPD) to the memory node. However, the reading is electronically scanned, and each row is read at a different time.

The Pinned Photodiode (PPD) is the largest component of the sensor. There are now two transfer gates: one to the memory node and one to the sensing node. The transfer gates are small in order to enable fast operation. The memory node is buried under the transfer gate (TX1) to mask it against stray photons. The Sensing Node (SN) is small in order to increase the analog gain.

The closing of the Global Shutter Reset (GRST) marks the start of the integration time, where the PPD is floating (not connected). The end of the integration time is achieved by operating the Global Transfer Gate (TX1) and transferring the signal electrons simultaneously to the memory node (MN). Prior to that, the MN is reset, as well as the SN. The reading is achieved by electronic scanning and is therefore row-dependent. The rows are scanned, and the second transfer gate is briefly operated, transferring the signal electrons to the SN node. Accordingly, the storage time of the memory node depends on the row. The reading of the signals with the source follower then follows exactly as in the four-transistor-APS (of Figure 1).

The value of the SN capacitance, C, also determines the kTC noise variance, which is contributed each time reset is performed. Earlier publications referred to hard reset and soft reset, arguing that, in the latter case, the noise is kTC/2. However, pixels designed with at least four transistors exhibit true correlated double sampling and cancel the kTC contribution.

Fossum overruled these arguments, indicating that the dominant noise is shot noise and not the thermal noise of the reset. The stochastic modelling presented below assumes that shot noise is the dominant noise and shows that the conversion gain obtained by the Mean-Variance method may yield either C/q or C/2q.

## 3. The New Stochastic Model

**Figure 3:**
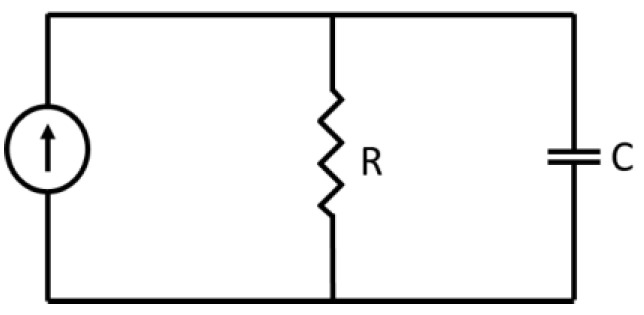
The electrical network model of the sensing junction.

Figure 3 shows the electrical network model for the sensing junction: a capacitor C in parallel with a resistance R, while, according to the Campbell–Hardy representation [29], an electrical current source is connected to the R-C network and injects the Shot-Noise current:

(2) $$i\left(t\right)=\sum_{k=-\infty }^{\infty }q_{0}\cdot \delta \left(t-T_{k}\right)$$

Here, $q_{0}$ is the electronic charge, $\delta \left(\cdot \right)$ is the Dirac Delta Function and ${\left\{T_{k}\right\}}_{k}$ is a train of mutually independent random variables, randomly distributed in time, with a constant average density λ per unit of time. Mathematically, $i\left(t\right)\;$ is a Poisson Point Process. The resulting average (i.e., dc current) of $i\left(t\right)\;$ is $I=q_{0}\cdot \lambda$, and the average value (i.e., dc) of the voltage over the capacitor is $V=IR=q_{0}\cdot \lambda \cdot R$.

The instantaneous voltage over the junction capacitor can be represented by a random process $v\left(t\right)$, which is related to the current $i\left(t\right)$. and to the electrical parameters R, C.

To calculate the variance of $v\left(t\right)$, one must deal with the (rather vague) interplay between a macroscopic measurement device and a bunch of individual electrons, randomly charging and discharging the junction capacitance. Putting this challenge in other words, we need to somehow characterize the random time-dependent reading of a macroscopic measuring device due to an individual shot-noise electron charging the junction capacitance at a random time $T_{k}$. The exact answer to this fundamental question of the interplay between a microscopic phenomenon and a macroscopic measuring device cannot be obtained, regardless of the detailed microscopic structure of the entire system, and this is, obviously, totally impractical. We can, however, tackle the problem by considering two rather extreme Model Cases.

### 3.1. Model Case I

In this model, we assume that the time dependence of the measuring device reading due to a shot-noise single electron current $q_{0}\cdot \delta \left(t-T_{k}\right)$ that charges the junction capacitance at a random time $T_{k}$ is:

(3) $$h\left(t\right)=\frac{q_{0}}{C}\cdot u\left(t\right)\cdot \exp\left(-\alpha \cdot t\right)$$

Here, $u\left(t\right)$ is the unit step function and α is a constant (which need not necessarily be equal to 1/RC, although this seems plausible). From (2) and (3) follows

(4) $$v\left(t\right)=\frac{q_{0}}{C}\cdot \overset{\infty }{\underset{k=-\infty }{\sum }}u\left(t-T_{k}\right)\cdot \exp\left(-\alpha \cdot [t-T_{k}]\right)$$

So, in this case, the voltage appears to be a pile-up of exponentially decaying pulses scattered randomly along the time axis.

According to the Campbell–Hardy theorem [29], the average of the random voltage (4), at any time t, is equal to:

(5) $$E\left\{v\left(t\right)\right\}=m_{v}=\lambda \overset{\infty }{\underset{-\infty }{\int }}h\left(t\right)dt=\lambda \cdot \frac{q_{0}}{C}\cdot \frac{1}{\alpha }$$

while the variance of that voltage, at any time t, is equal to:

(6) $$E\left\{{ [v\left(t\right)-m_{v}]}^{2}\right\}=\sigma_{v}^{2}=\lambda \overset{\infty }{\underset{-\infty }{\int }}h^{2}\left(t\right)dt=\lambda \cdot {\left(\frac{q_{0}}{C}\right)}^{2}\cdot \frac{1}{2\alpha }$$

Hence, according to Model Case I, the ratio between the variance and average is:

(7) $$\frac{\sigma_{v}^{2}}{m_{v}}=\frac{q_{0}}{2C}$$

### 3.2. Model Case II

In this second model, we assume that the time dependence of the measuring device reading due to a shot-noise single electron current $q_{0}\cdot \delta \left(t-T_{k}\right)$ that charges the junction capacitance at a random time $T_{k}$ is a rectangular voltage pulse that appears at the random time $T_{k}$ and lasts for a random time duration $X_{k}$, namely,

(8) $$h_{k}\left(t-T_{k}\right)=\frac{q_{0}}{C}\cdot [u\left(t-T_{k}\right)-u\left(t-T_{k}-X_{k}\right)]$$

It follows that, in this case, $v\left(t\right)$ is a sum of rectangular voltage pulses that appear at independent random time instances ${\left\{T_{k}\right\}}_{k}$ and have independent random durations ${\left\{X_{k}\right\}}_{k}$, explicitly:

(9) $$v\left(t\right)=\frac{q_{0}}{C}\cdot \overset{\infty }{\underset{k=-\infty }{\sum }} [u\left(t-T_{k}\right)-u\left(t-T_{k}-X_{k}\right)]$$

As different shot-noise electrons are physically identical, all the random variables ${\left\{X_{k}\right\}}_{k}$ have identical probability qualities, obey the same probability density function and, in particular, have an identical average value $m_{X}$; hence, $m_{X}=E\left\{X_{k}\right\}$ for all k.

We now note that, since $X_{k}\geq 0$, it follows that:

(10) $$h_{k}\left(t\right)=u\left(t\right)-u\left(t-X_{k}\right)={ [u\left(t\right)-u\left(t-X_{k}\right)]}^{2}=\left\{\begin{array}{l} 1,\;\;0\leq t\leq X_{k}\\ 0,\;\;t\geq X_{k} \end{array}\right.$$

and, therefore:

(11) $$E\left\{\overset{\infty }{\underset{-\infty }{\int }}h_{k}\left(t\right)dt\right\}=E\left\{\overset{\infty }{\underset{-\infty }{\int }} [u\left(t\right)-u\left(t-X_{k}\right)]dt\right\}=E\left\{X_{k}\right\}=m_{X}$$

and, likewise:

(12) $$E\left\{\overset{\infty }{\underset{-\infty }{\int }}h_{k}^{2}\left(t\right)dt\right\}=E\left\{\overset{\infty }{\underset{-\infty }{\int }}{ [u\left(t\right)-u\left(t-X_{k}\right)]}^{2}dt\right\}=E\left\{X_{k}\right\}=m_{X}$$

Hence, the Campbell–Hardy theorem implies that, in Model Case II, the average and variance of $v\left(t\right)$ at any time t are, respectively,

(13) $$E\left\{v\left(t\right)\right\}=m_{v}=\lambda \cdot E\left\{\overset{\infty }{\underset{-\infty }{\int }}h_{k}\left(t\right)dt\right\}=\lambda \cdot \frac{q_{0}}{C}\cdot m_{X}$$

and

(14) $$E\left\{{ [v\left(t\right)-m_{v}]}^{2}\right\}=\sigma_{v}^{2}=\lambda \cdot E\left\{\overset{\infty }{\underset{-\infty }{\int }}h_{k}^{2}\left(t\right)dt\right\}=\lambda \cdot {\left(\frac{q_{0}}{C}\right)}^{2}\cdot m_{X}$$

So, in Model Case II, the variance–average ratio is:

(15) $$\frac{\sigma_{v}^{2}}{m_{v}}=\frac{q_{0}}{C}$$

which is twice the ratio obtained previously with Model Case I.

In this study, we apply both models to analyze the measured results. Model Case I allows us to obtain consistent results between the measurements and the simulation.

## 4. Measurement Results

The measurements taken include Conversion Gain (CG), later used for Quantum Efficiency (QE) measurements. CG is measured by the Mean-Variance method under illumination.

**Figure 4:**
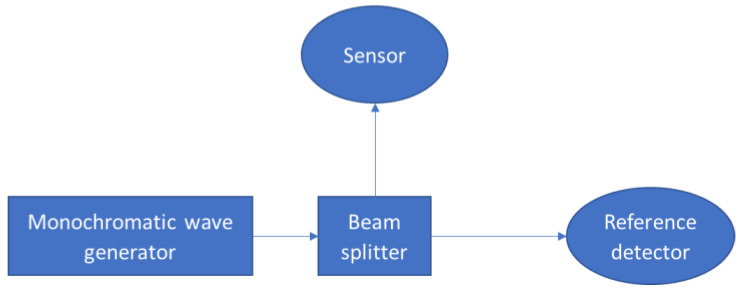
The measurement setup for optical characterization.

The measurement setup is shown in Figure 4.

The system that was used in this experiment was an external quantum efficiency (EQE, Newport, RI, USA). The light source was a Quartz Tungsten Halogen (QTH) lamp which was put through a CS260 Monochromator (step size of 1 nm at a spectral range of 400–1100 nm). The incident beam was directed onto a beam splitter that projected the light simultaneously onto the device under study (DUT) and onto a 918D calibrated power meter (Newport USA). Both devices were carefully mounted at the same distance from the center of the beam splitter (4 cm).

**Figure 5:**
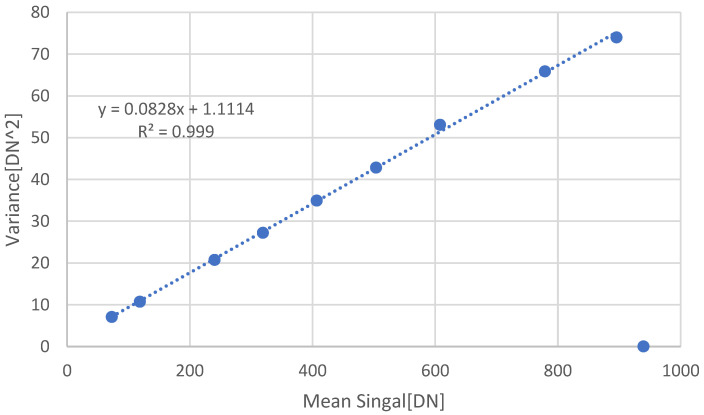
Conversion Gain measurements using the Mean-Variance method under illumination at 550 nm. The slope is 0.0828 [DN/e], leading to a Conversion Gain value of 6 [e/DN] using Model Case I.

The monochromator generated a beam at the wavelength of 550 nm. A total of 100 frames were taken from the DUT sensor, from which the mean and variance were extracted. Figure 5 shows the measurement results under illumination. The slope is 0.0828 [DN/e]. The Conversion Gain extracted from the measurements using Model Case I is 6 [e/DN], and, using Model Case II, it is 12 [e/DN].

If we look at the reciprocal of the CG and include the new factor of 2, we obtain the Conversion Gain in terms of [e/DN]. The slope extracted from the graph is 0.0828 [DN/e]. According to Model Case I, half of the slope value should be used: slope/2 = 0.0828 [DN/e]. Therefore, slope = 0.1656 [DN/e]. The reciprocal value of the slope is CG = 6 [e/DN].

### From Photons to Bits: Responsivity and Quantum Efficiency Times Fill Factor (FF)

The quantum efficiency of an imager is defined as the number of electrons generated in each pixel per incident photon on that pixel under steady-state conditions. In practice, the electrons are collected by the PPD, while the photons are irradiated on the pixel. Hence, what we measure (and simulate) is the QE*FF, where the Fill Factor is the ratio of the PPD area to the pixel area.

To determine the photo response (responsivity in Amp/Watt) and quantum efficiency, two parameters are required in addition to the Conversion Gain and Fill Factor:

(1) The incident input optical power, which can be calculated using a reference calibrated photodetector for a particular wavelength.
(2) The output electrical signal of the DUT, which is determined by measuring the output signal over a fixed integration time. Hence, at a given wavelength and for a given input optical power, one can evaluate the photo response.

For each wavelength, the monochromatic power flux is measured with the reference detector in units of Watt/cm^2^ (assuming an aperture of 1 cm^2^, as specified by the vendor). We assume that, with the beam splitter, the DUT is irradiated with the same power flux. A total of 100 frames of the array were taken for each wavelength. The dark frame was subtracted from those frames. The signal illumination level mean value was calculated per each pixel over the frames. Then, the pixel mean values were averaged, making sure that the light intensity did not saturate the pixel measurements:

(16) $$\bar{S_{i,j}}=\frac{\sum_{k}S_{i,j,k}}{N}\;\;;\;\bar{S}=\frac{\sum_{i,j}\bar{S_{i,j}}}{ML}$$

where *S* is the signal illumination level in DN, *k* is the frame index, *N* is the number of frames, *i* is the row index, *j* is the column index, *M* is the number of rows in the array and *L* is the number of columns in the array.

For a measurement integration time of τ, the pixel is irradiated with a photons flux, which can be evaluated by assuming the pixel area.

**Figure 6:**
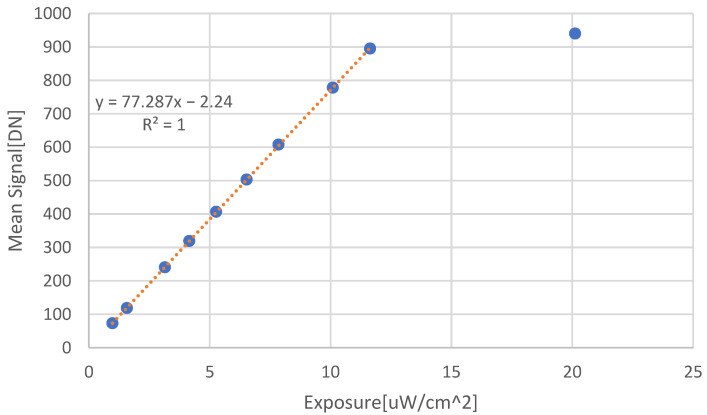
Measured data from which the responsivity of the sensor is extracted in units of [A/Watt], at 550 nm.

The incident optical power is varied to show the linearity and to obtain the responsivity (see Figure 6):

The responsivity extracted from Figure 6, according to:

(17) $$R [\frac{A}{W}]=\underset{Slope}{\underbrace{\frac{Mean\;Signal [DN]}{Exposure [\frac{W}{cm^{2}}]}}}\cdot \frac{CG [\frac{e}{DN}]\cdot q_{0} [Cb]}{A_{sensor} [cm^{2}]\cdot \tau [s]}$$

where $q_{0}$ is the electron charge, and $A_{sensor}$ is the sensor area.

Assuming an integration time of 1.43 [ms] and $A_{sensor}=28.8\cdot 10^{-8} [cm^{2}]$, the resulting responsivity is R = 0.1801 [A/W].

QE can then be extracted by the responsivity definition:

(18) $$R [\frac{A}{W}]=\frac{I}{P_{opt}}=\frac{q_{0}\eta N_{0}A_{PPD}}{\left(\frac{hc}{\lambda }\right)N_{0}A_{sensor}}=\left(\frac{\lambda }{hc}\right)q\eta \cdot FF$$

where *N*_0_ is the number of photons per time, *η* represents the QE of the sensor, $A_{PPD}$ is the PPD area, *h* is the Planck constant and *c* is the speed of light. The ratio between the PPD area and the sensor area is the Fill Factor of the sensor (FF). In our case, micro-lenses are applied on the sensor, resulting in an FF of approximately 1. Using that calculation method, the QE at 550 nm reached 40.7%.

## 5. Simulation

The pixel’s optical modeling and simulations were carried out using LUMERICAL [26] software. The QE was simulated assuming that epilayer effective thickness is 9 μm and that there are micro-lenses. The absorbed power under the PPD is divided by the total illuminated power.

The modeling did not include electrical aspects, assuming every absorbed photon resulted in a measured electron. Therefore, the simulation results were expected to be higher than the measurements.

**Figure 7:**
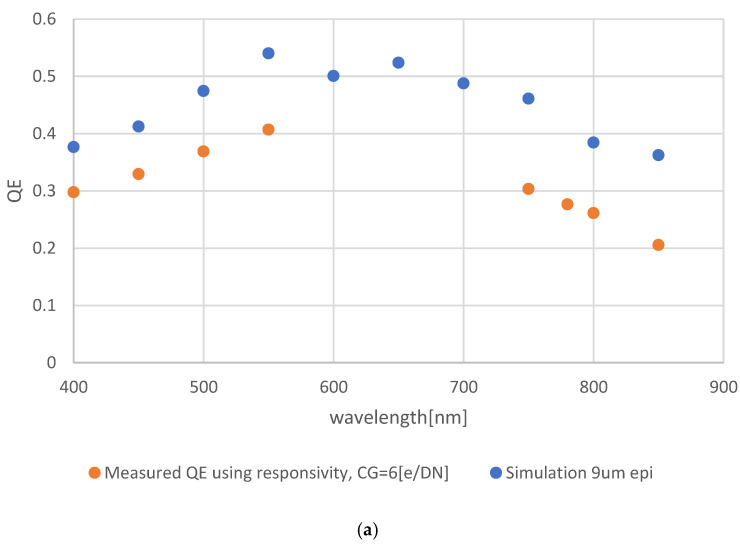

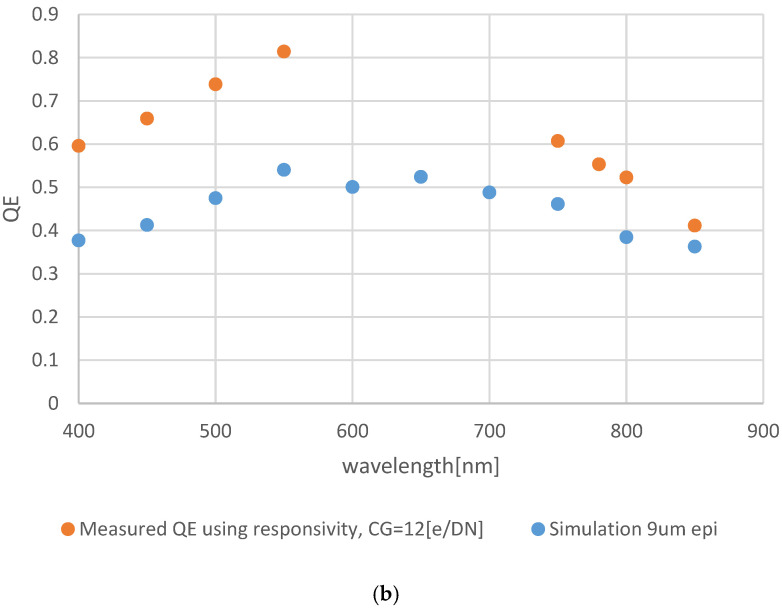
(**a**) Measured and Simulated QE using Model Case I of Section 3 for CG modeling, with the value of CG = 6 [e/DN]. (**b**). Measured and Simulated QE using Model Case II (Section 3), with the value of CG = 12 [e/DN].

Figure 7a,b show the comparison between the measurements and the simulation using the first and second CG Model Cases, respectively.

From the comparisons, the QE using Model Case I provides reasonable values that match the simulation. As expected, the simulation is quite higher than the measurements using Model Case I. By using Model Case II, the simulated results are lower than the measured results, which is non-physical. During measurements, not all photo-carriers are collected by the PPD, and, hence, the simulated results should be higher.

## 6. Summary and Conclusions

This study presents a new stochastic model for the Conversion Gain. The model is based on fundamental physical assumptions: the dominant noise of the pixel in a well-designed imager is shot noise. The model revisits the “gold standard” method of the measured variance of the shot noise to the mean value. The random time-dependent of any shot-noise electron charging the junction capacitance may have either an exponential form or a step form. In the former case, a factor of 1/2 appears in the variance to the mean value, namely, q/2C is obtained. In the latter case, the well-established ratio q/C remains. The model is microscopic, while the measurement is macroscopic. The two limiting effects are different by a factor of 2, which is significant for the determination of the conversion gain. Therefore, the conversion gain, which translates the digital numbers DN into a physical entity (electrons), should be validated.

The validation may be achieved by evaluating the quantum efficiency and by comparing the results to simulations and physical modeling. All the parameters should fit. The measured quantum efficiency should be less than the optically simulated results. The physical modeling and the simulation require an understanding of the pixel design as well as the readout. Hence, contrary to the prevailing concept, one cannot treat the sensor as a black box.

In summary, this study is based on the earlier, excellent and extensive work reported in the literature by many scientists and engineers. This is why the authors framed the paper as “revisiting” and not “revising” the conversion gain. The author’s main message is that characterizing advanced digital imagers is challenging. By better understanding all the aspects of the system, including the pixel layout, the analog part and the digital part, errors may be avoided, and the generic design may be optimized for each use case.

## Data Availability

Not applicable.
